# High-TSH Subclinical Hypothyroidism Is Associated With Postoperative Mortality in Acute Type A Aortic Dissection

**DOI:** 10.3389/fendo.2022.844787

**Published:** 2022-04-29

**Authors:** Shi-Pan Wang, Yuan Xue, Hai-Yang Li, Wen-Jian Jiang, Hong-Jia Zhang

**Affiliations:** ^1^Department of Cardiac Surgery, Beijing Anzhen Hospital,Capital Medical University, Beijing, China; ^2^Beijing Institute of Heart, Lung and Blood Vessel Diseases, Beijing, China; ^3^Beijing Advanced Innovation Center for Big Data-based Precision Medicine, Capital Medical University, Beijing, China; ^4^Beijing Lab for Cardiovascular Precision Medicine, Beijing, China; ^5^Key Laboratory of Medical Engineering for Cardiovascular Disease, Beijing, China

**Keywords:** subclinical hypothyroidism, thyroid-stimulating hormone, acute type A aortic dissection, total arch replacement, postoperative mortality

## Abstract

**Background:**

Subclinical hypothyroidism can negatively affect the cardiovascular system and increase the risk of mortality, especially for individuals with thyroid-stimulating hormone (TSH) levels above 10 mU/L. We investigated the relationship between high-TSH subclinical hypothyroidism and postoperative mortality in acute type A aortic dissection (ATAAD) patients.

**Method:**

We enrolled 146 patients with ATAAD who underwent aortic surgery in Beijing Anzhen Hospital from July 2016 to November 2018. Thyroid hormone levels were obtained before surgery, and participants were divided into a ≥10mU/L TSH level group and a <10mU/L level group. Cox proportional hazard regression and subgroup analysis were conducted to examine the association of preoperative high-TSH subclinical hypothyroidism with postoperative mortality.

**Result:**

Participants with preoperative high-TSH (≥10mU/L) subclinical hypothyroidism tended to have longer hospitalization stays after surgery [16.0 (IQR 11.0-21.0) days vs 12.5 (IQR 8.0-16.0) days, P=0.001]. During the first 30 days after operation, 15 of 146 patients died (10.3%); during a median of 3.16 (IQR 1.76-4.56) years of follow-up, 24 patients died (16.4%). Cox proportional hazard regression showed that preoperative high-TSH subclinical hypothyroidism was independently associated with 30-day mortality (HR=6.2, 95% CI, 1.7-22.0, P=0.005) and postoperative mortality after adjusting for age, sex, BMI, hypertension, ejection fraction, diabetes and history of PCI (HR=3.4, 95% CI, 1.4-8.0, P=0.005).

**Conclusion:**

This study showed that preoperative high-TSH subclinical hypothyroidism was an independent predictor of postoperative mortality in ATAAD patients who underwent aortic surgery.

## Introduction

Subclinical hypothyroidism (SCH) is defined as elevated thyroid-stimulating hormone (TSH) with a normal level of free thyroxine (FT4) (1). Mostly, SCH is harmless, therefore, most individuals do not receive any treatment. However, for individuals with TSH concentration above 10 mU/L, the risk of cardiovascular disease is high (2). Some meta-analyses have revealed that SCH is related to increased risk of cardiovascular mortality and congestive heart failure in subjects with TSH concentrations ≥10 mU/L (3, 4). Under these conditions, significant increase in TSH may lead to adverse outcomes.

Acute type A aortic dissection (ATAAD) is a devastating disease that requires urgent surgical intervention according to current guidelines (5). Although rapid surgical intervention rescues more patients with ATAAD, the operative mortality is still high. The 30-day mortality is between 10%-35%, and 5-year survival rate is 63% (6). Preoperative malperfusion, high level of N-terminal pro-brain natriuretic peptide, history of cardiac disease, and cardiopulmonary resuscitation may predict adverse events (7–9). However, the relationship between preoperative SCH and postoperative outcomes has not been clarified. Therefore, we investigated a possible association between SCH and postoperative death of patients with ATAAD.

## Materials and Methods

### Patient Selection

We enrolled consecutive patients who were admitted to Beijing Anzhen Hospital because of acute Type A aortic dissection (ATAAD) from July 2016 to November 2018. We excluded individuals with one or more of the following factors: (1) genetic syndrome related to aortic disease, such as Marfan, Loeys–Dietz, Turner, or Ehlers–Danlos syndrome; (2) history of thyroid disease, pituitary diseases and administration of drugs that affect thyroid function; (3) disease that affects thyroid binding protein concentration, such as dysfunction of liver, pregnancy, and malignant tumors. Finally, 146 ATAAD patients were included for analysis.

### Data Collection

Baseline data included age, body mass index (BMI), sex, tobacco use, diabetes, hypertension, cardiac disease, cerebral infarction, coronary heart disease, previous thoracic endovascular aortic repair, previous percutaneous coronary intervention (PCI), preoperative creatinine concentration, ascending aorta diameter and left ventricular ejection fraction. Intraoperative variable was composed of nasopharyngeal temperature, anal temperature (the lowest temperature recorded during cardiopulmonary bypass), cardiopulmonary bypass time, deep hypothermia circulatory arrest time, aortic cross-clamp time, operation time, and the type of operation, including ascending aorta replacement, aortic root replacement, partial arch replacement, Sun’s procedure, coronary artery bypass grafting, and mitral valve surgery. Postoperative information included hospitalization days, ventilator time, intensive care unit retention time, neurological complications, cardiovascular complications, respiratory complications, and postoperative dialysis; this information was assembled from Electronic Medical Records.

### Surgical Management

The same surgical strategies were adopted for ATAAD as described (10). All surgical procedures were performed by a median sternotomy and total cardiopulmonary bypass (CPB) with selective cerebral perfusion (SCP). The right axillary artery was used for cannulation during the CPB and SCP. Total arch replacement (TAR) with the frozen elephant trunk technique was performed with the patient under hypothermic circulatory arrest and unilateral anterior selective cerebral perfusion through the right axillary artery, with a flow rate of 5-10 ml/kg per min and a perfusion pressure of 40-60 mmHg. The nasopharyngeal temperature was lowered to 22°C to 28°C.

### End Points and Definitions

The primary end point of this study was postoperative mortality. Thirty-day mortality was defined as death occurring by any cause during the first 30 days after surgery or as death in hospital after surgery. Subclinical hypothyroidism was defined as elevated TSH with a normal concentration of free thyroxine (FT4) (1). On the basis of previous research, SCH patients with serum TSH concentrations of 10 mU/L or greater have high cardiovascular risk (2–4). Thus, we divided the 146 participants into two groups according to serum TSH concentration, ≥10mU/L and <10mU/L. All patients were diagnosed with acute type A aortic dissection (ATAAD) by computed tomography angiography. ATAAD was defined as the dissection involving ascending aorta with presentation of symptom onset less than 14 days according to the current guideline (5). Neurological complications were composed of cerebral hemorrhage, cerebral infarction, limb paralysis and paraplegia. Cardiovascular complication was defined as cardiac tamponade, arrhythmia and use of Intra-Aortic Balloon Pump. Respiratory complications included lung infection, pleural effusion, and respiratory failure. Acute Kidney Injury was defined by an increased plasma creatinine concentration within one week according to the Kidney Disease: Improving Global Outcomes (KDIGO) classification (11).

### Blood Sample Analysis

After collection, the blood sample was anticoagulated with sodium citrate and centrifuged for 15 min at 3,500 rpm at 4°C. All the samples were divided into 0.5-ml centrifuge tubes, stored at−80°C, and used only for this study. The preoperative plasma concentration of thyroid hormone was determined by ELISA (Uscn, Wuhan, China), as described (12). The mean sample storage duration was 84.5 days when ELISA was performed. All ELISAs were performed twice, and the mean value was used for analysis.

### Statistical Analysis

**Figure 1:**
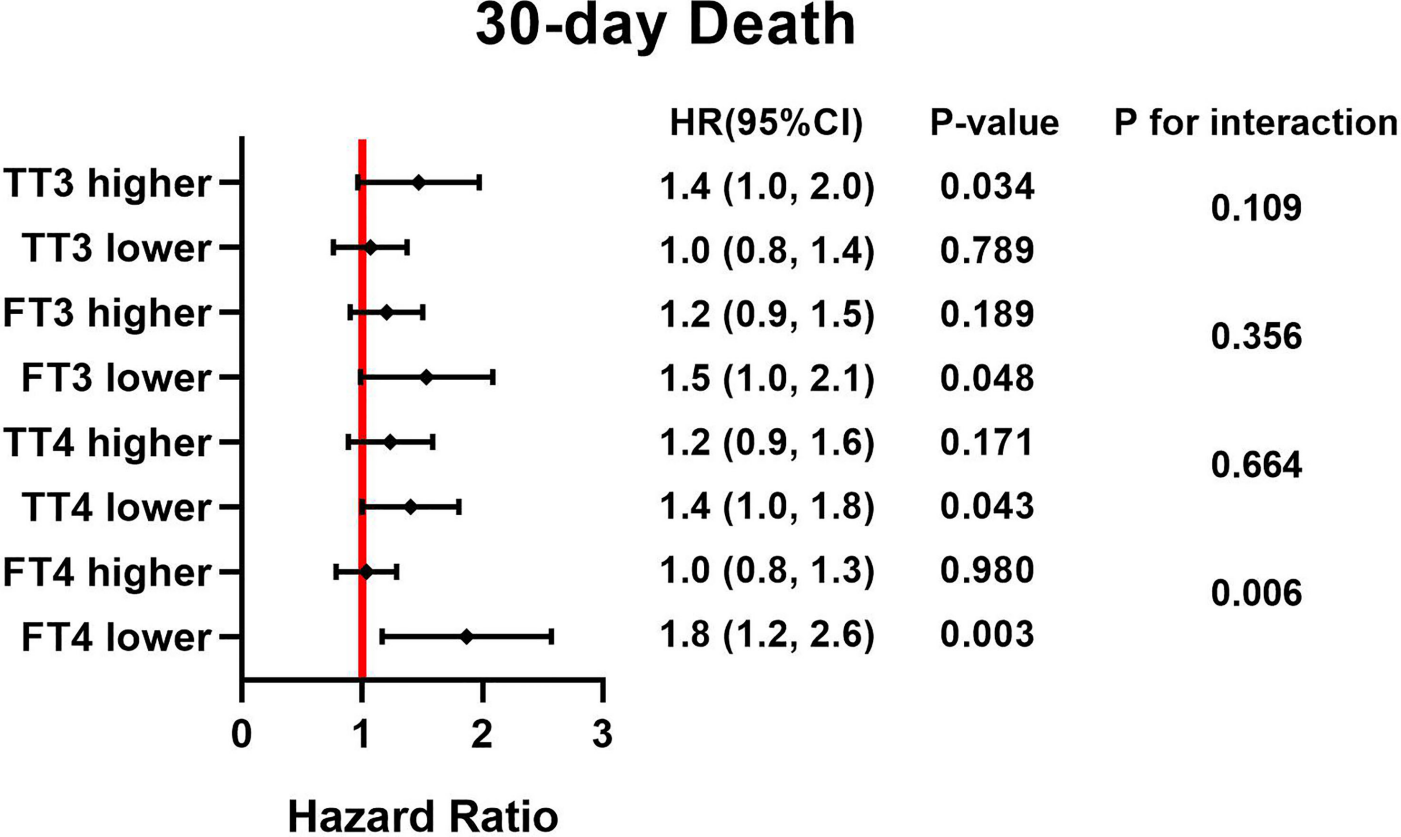
HRs for TSH and 30-day death based on different thyroid hormone concentration.

**Figure 2:**
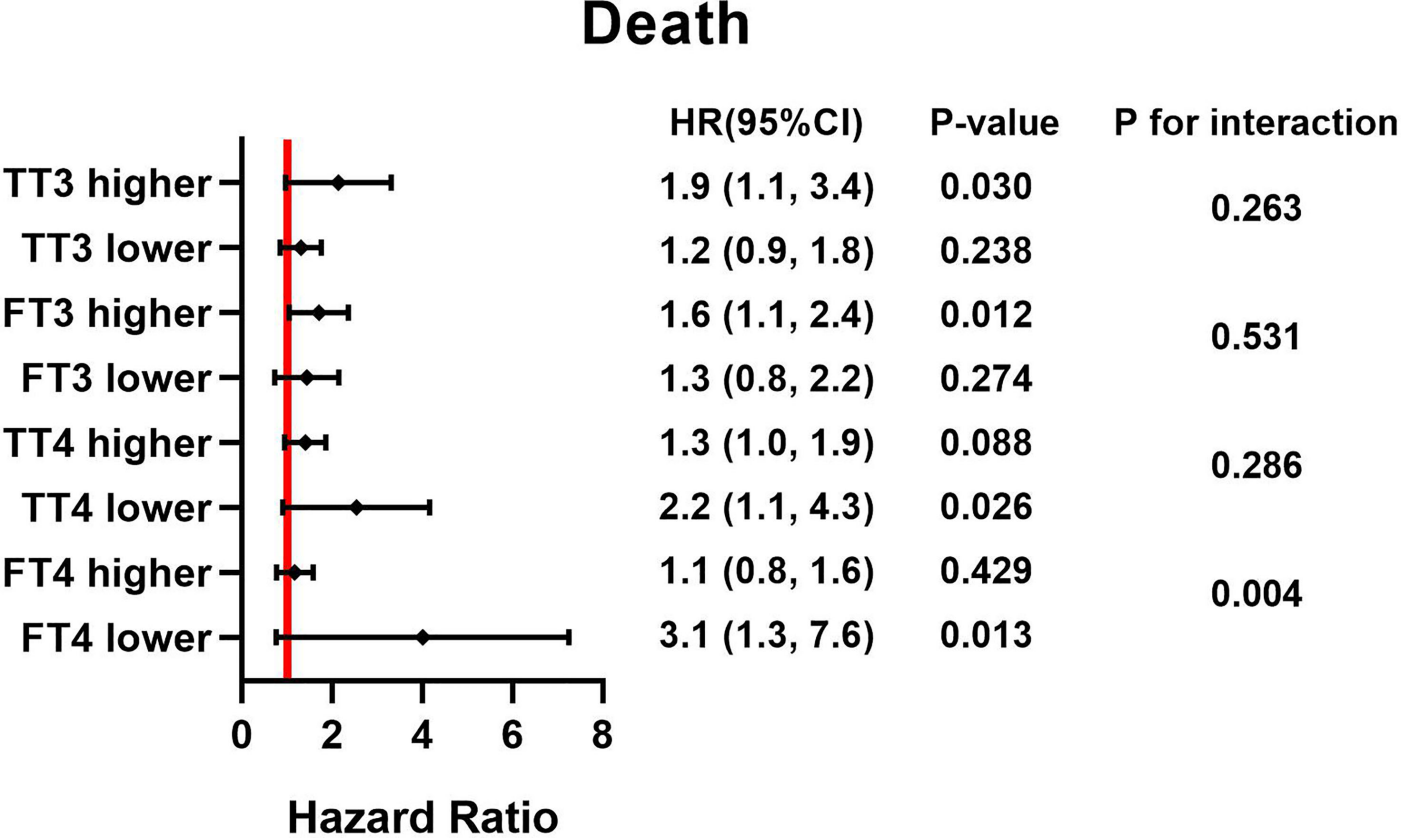
HRs for TSH and postoperative death based on different thyroid hormone concentration. Adjust for, Age; Sex; BMI. HR, hazard ratio; BMI, body mass index; TSH, thyroid stimulating hormone; TT3, total triiodothyronine; FT3, free triiodothyronine; TT4, total thyroxine; FT4, free thyroxine.

Continuous variables were expressed as mean ± standard deviation or median with interquartile range according to their normality. Continuous variables were compared using the unpaired Student’s t-test or Mann-Whitney U-test as appropriate. One-way ANOVA and the Kruskal-Wallis test were used for normally continuous variables and skewed continuous variables, respectively. Categorical variables are shown as actual numbers or percent and were compared using the Pearson’s Chi square test or Fisher’s exact test as appropriate. Cox proportional hazard regression analyses were performed to identify the independent predictive ability of TSH and postoperative death. We present regression results only as (1) unadjusted model, (2) model adjusted for age and sex, and (3) model adjusted for age, sex, BMI, hypertension, ejection fraction, diabetes and history of PCI. In the subgroup analyses, the different types of thyroid hormone were divided into higher and lower categories by their median, and we conducted stratification analyses to examine whether the effects of the TSH concentration differed across various subgroups. Their interactions were tested simultaneously and demonstrated by a Forest plot (**Figures 1**, **2**). We calculated the survival rate by using the Kaplan-Meier analytical method, and patients were categorized by different concentrations of TSH; differences between groups were estimated by using the log-rank test. A two-tailed P value <0.05 was considered statistically significant. All of the analyses were performed with the statistical software packages R (http://www.R-project.org, The R Foundation).

## Results

### Baseline Characteristics

We included 146 patients in our analysis. Sixty patients had a TSH value of 10mU/L or greater, and all the patients had a normal concentration of FT4. **Table 1** shows the preoperative baseline characteristics. The mean age in the cohort was 49.5 ± 11.6 years old; there were 38 women (26.0%). **Table 2** lists details from during and after the operation. We observed longer hospitalization time in the TSH ≥10 mU/L group with significant statistical differences [16.0 (IQR 11.0-21.0) days vs 12.5 (IQR 8.0-16.0) days, P=0.001]. However, there were no other statistical differences in preoperative and postoperative characteristics between the ≥10 mU/L and <10mU/L groups.

**Table 1:** Baseline characteristics of participants.

Patient demographics	Total (N = 146)	TSH category		*P-value*
		<10 mU/L (N = 86)	≥10 mU/L (N = 60)	
Age, years	49.5 ± 11.6	49.4 ± 11.4	49.6 ± 12.0	0.926
BMI, kg/m^2^	25.9 ± 3.9	26.0 ± 3.9	25.8 ± 3.8	0.793
Sex (Female)	38 (26.0%)	24 (27.9%)	14 (23.3%)	0.535
Smoking	64 (43.8%)	37 (43.0%)	27 (45.0%)	0.813
Diabetes	9 (6.2%)	6 (7.0%)	3 (5.0%)	0.625
Hypertension	98 (67.1%)	57 (66.3%)	41 (68.3%)	0.795
Cardiac disease	21 (14.4%)	13 (15.1%)	8 (13.3%)	0.763
Cerebral infarction	10 (6.8%)	6 (7.0%)	4 (6.7%)	0.942
Coronary heart disease	9 (6.2%)	6 (7.0%)	3 (5.0%)	0.625
TEVAR	6 (4.1%)	4 (4.7%)	2 (3.3%)	0.693
PCI	4 (2.7%)	3 (3.5%)	1 (1.7%)	0.507
Preoperative creatinine, μmol/L	93.2 ± 91.3	87.1 ± 34.2	102.1 ± 137.3	0.333
Ascending aorta diameter, mm	46.4 ± 8.3	46.4 ± 8.6	46.5 ± 7.9	0.960
LVEF, %	61.9 ± 6.2	61.4 ± 6.2	62.6 ± 6.2	0.291

Results are expressed as n (%) or mean ± standard deviation or median interquartile range.

BMI, body mass index; TEVAR, thoracic endovascular aortic repair; PCI, Percutaneous coronary intervention, LVEF, left ventricular ejection fraction.

**Table 2:** Surgical details and postoperative outcomes.

Operative details	Total (N = 146)	TSH category		*P-value*
		<10 mU/L (N = 86)	≥10 mU/L (N = 60)	
Nasopharyngeal temperature, °C	24.5 ± 2.3	24.5 ± 2.2	24.5 ± 2.5	0.912
Anal temperature, °C	26.0 ± 2.2	26.2 ± 2.0	25.8 ± 2.5	0.359
Cardiopulmonary bypass time, min	193.8 ± 57.6	188.3 ± 49.8	201.6 ± 67.0	0.171
Deep hypothermic circulatory arrest time, min	21.4 ± 11.8	20.7 ± 11.8	22.4 ± 11.7	0.390
Aortic cross-clamp time, min	112.1 ± 38.0	109.8 ± 35.8	115.4 ± 41.2	0.384
Operation time, hours	7.4 ± 1.7	7.2 ± 1.6	7.7 ± 1.7	0.060
Ascending aorta replacement	85 (58.2%)	48 (55.8%)	37 (61.7%)	0.481
Aortic root replacement	60 (41.1%)	37 (43.0%)	23 (38.3%)	0.571
Partial arch replacement	20 (13.7%)	12 (14.0%)	8 (13.3%)	0.915
Sun’s procedure	119 (81.5%)	71 (82.6%)	48 (80.0%)	0.695
Coronary artery bypass grafting	8 (5.5%)	6 (7.0%)	2 (3.3%)	0.341
Mitral valve repair/replacement	6 (4.1%)	4 (4.7%)	2 (3.3%)	0.693
Postoperative outcomes				
Hospitalization days, days	14.0 (10.0-19.0)	12.5 (8.0-16.0)	16.0 (11.0-21.0)	0.001
Ventilator time, hours	30.0 (17.2-76.0)	24.5 (16.0-64.4)	32.0 (19.0-90.2)	0.671
Intensive care unit retention time, hours	37.0 (18.0-87.0)	33.5 (17.0-81.2)	41.0 (19.0-104.5)	0.398
Neurological complications	15 (10.3%)	10 (11.6%)	5 (8.3%)	0.519
Cardiovascular complication	34 (23.3%)	18 (20.9%)	16 (26.7%)	0.420
Respiratory complication	52 (35.6%)	29 (33.7%)	23 (38.3%)	0.567
Postoperative dialysis	15 (10.3%)	9 (10.5%)	6 (10.0%)	0.927

Results are expressed as n (%) or mean ± standard deviation or median interquartile range.

### Univariate and Multivariate Cox Regression Analyses

As shown in **Table 3**, it appeared that high-TSH SCH was significantly associated with 30-day mortality (HR 6.3, 95% CI 1.8-22.4, P=0.004), and the incidence of postoperative mortality remained significantly high in high-TSH SCH patients (HR 3.2, 95% CI 1.4-7.5, P=0.007).

**Table 3:** Cox-Regression analyses of hazard ratios in high-TSH subclinical hypothyroidism associated with 30-day mortality and postoperative mortality in patients with ATAAD.

30-day Death			
Variable	HR	95% CI	P-value
Crude Model	6.3	1.8, 22.4	0.004
Model I	6.5	1.8, 23.0	0.004
Model II	6.2	1.7, 22.0	0.005
**Death**			
**Variable**	**HR**	**95% CI**	**P-value**
Crude Model	3.2	1.4, 7.5	0.007
Model I	3.4	1.4, 7.9	0.005
Model II	3.4	1.4, 8.0	0.005

Crude model adjusted: None.

Model I adjusted for: Age; Sex.

Model II adjusted for: Age; Sex; BMI; Hypertension; Ejection Fraction; Diabetes; History of PCI

HR: hazard ratio; CI: confidence interval.

After adjusting for age, sex, BMI, hypertension, ejection fraction, diabetes and history of PCI, we observed that the risk of both 30-day mortality and postoperative mortality were still significant in high-TSH SCH patients. (HR 6.2, 95% CI 1.7-22.0, P=0.005; HR 3.4, 95% CI 1.4-8.0, P=0.005, respectively).

### Subgroup Analysis

Forest plots (**Figures 1**, **2**) demonstrate the influence of TSH on 30-day and postoperative mortality which was binary stratified by preoperative total triiodothyronine (TT3), free triiodothyronine (FT3), total thyroxine (TT4) and FT4 levels. The cutoff value of each group is as followed: 63.953 pmol/L for TT3, 7.684 pmol/L for FT3, 97.185 nmol/L for TT4, and 16.979 pmol/L for FT4. Notably, TSH concentration was significantly related to the risk of 30-day and postoperative mortality, which was observed across different thyroid hormone concentrations. Moreover, there was a significant interactive effect of FT4 and TSH concentrations on 30-day mortality and postoperative mortality. (P for interaction = 0.006 and 0.004, respectively).

### Kaplan-Meier Survival Analyses

**Figure 3:**
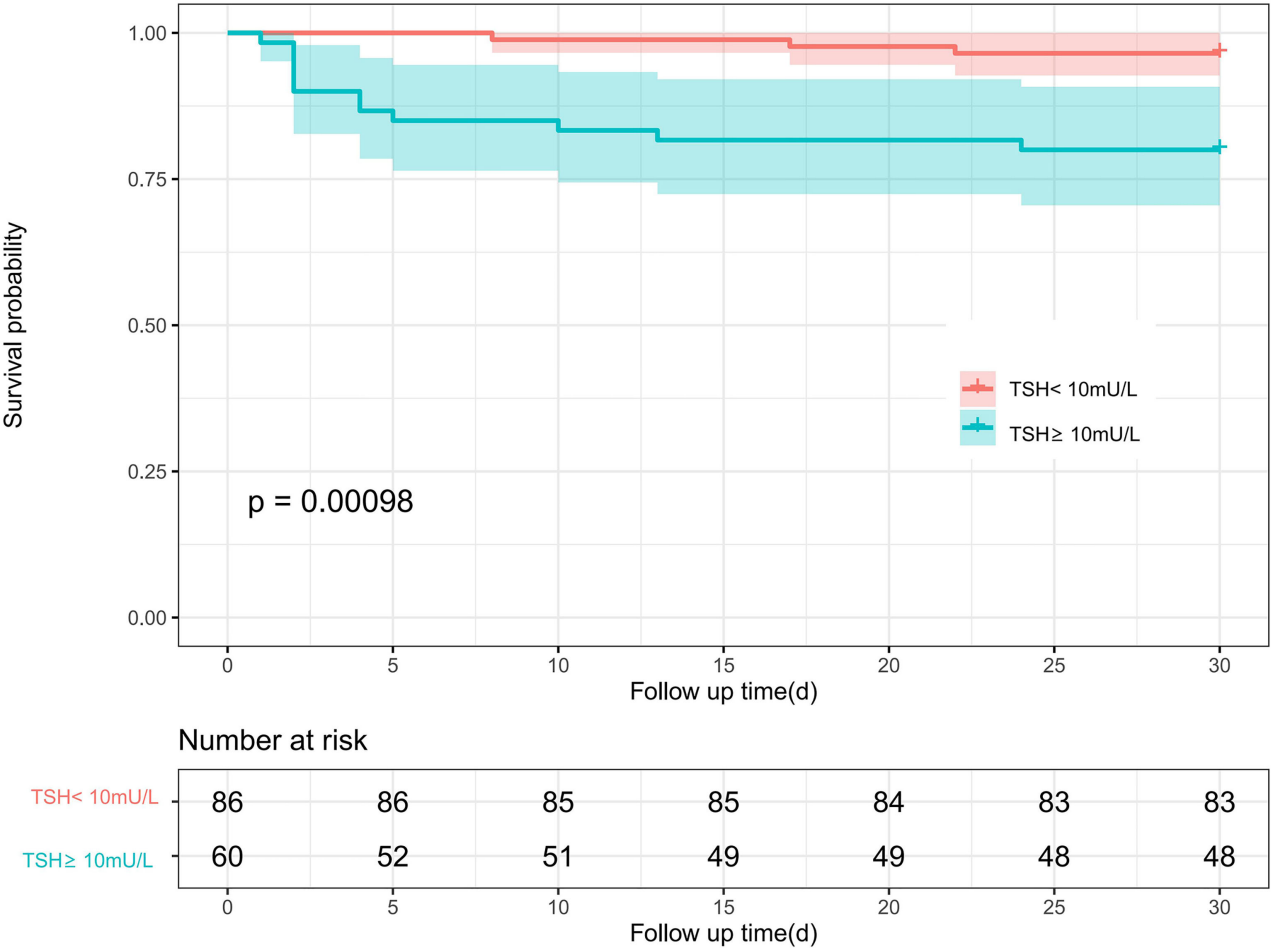
Kaplan–Meier curve of different TSH level with 30-day mortality among ATAAD patients.

**Figure 4:**
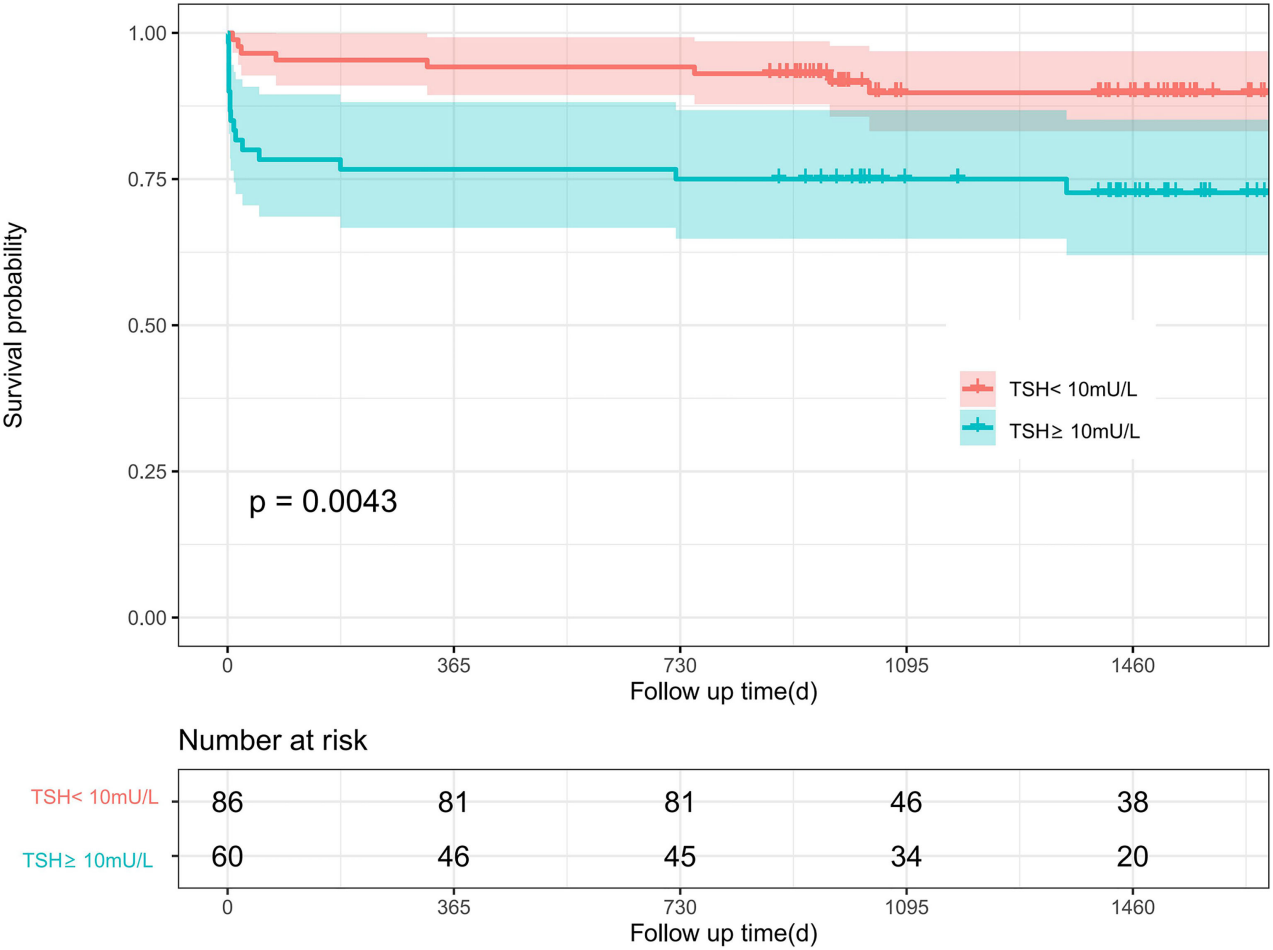
Kaplan–Meier curve of different TSH level with postoperative mortality among ATAAD patients. Log-rank P values are shown. TSH, thyroid stimulating hormone. 95% confidence intervals are shown in red and blue shadows.

During the first 30 days after the surgical procedure, 15 patients died (3 cases in TSH<10mU/L group, 12 cases in TSH≥10mU/L); during a median of 3.16 (IQR 1.76-4.56) years of follow-up, 24 patients died (8 cases in TSH<10mU/L group, 16 cases in TSH≥10mU/L). The Kaplan-Meier curves (**Figures 3**, **4**) demonstrate a significant correlation of TSH with 30-day and postoperative mortality when we categorized TSH into ≥10mU/L and <10mU/L groups (Log-rank P<0.001 and P<0.01, respectively).

## Discussion

Previously, elevated TSH level in blood was a predictor of worse outcomes, such as increased mortality among the critical patients in ICU (13) and prolonged hospital stay, and this may be due to the effects of hypothyroidism, such as increased systemic vascular resistance, decreased cardiac contractility, decreased cardiac output and accelerated atherosclerosis and coronary artery disease, the mechanism can be complexed (14). Chen et al. (15) found that even a mild increase in TSH concentration (4.5-9.9 mU/L) was associated with reduced survival compared with a normal TSH concentration among heart failure patients. However, numerous studies have focused on the impact of subclinical thyroid dysfunction in cardiovascular disease. In 2012, Tseng and colleagues (16) reported that SCH was associated with increased cardiovascular disease mortality. Moreover, the risk of cardiac events depends on the severity of hormonal dysfunction, tending to be higher when TSH≥7.0 mU/L and even more evident when the value≥10 mU/L (3). Likely, in a large-scaled meta-analysis with 1,898,314 participants from 55 cohort studies (17), SCH with TSH level ≥10mU/L was especially associated with high risks of ischemic heart disease.

Preoperative SCH can be observed in patients who undergo cardiac surgery such as coronary artery bypass grafting (CABG) or heart valve surgery, leading to poor prognosis (18). Nevertheless, whether the severity of SCH is associated with ATAAD patient adverse outcomes has not been confirmed. Therefore, we designed this retrospective study to clarify the relationship.

Among our participants, 60 of 146 had TSH concentration ≥10 mU/L (41.1%). Among the sixty patients, 16 died after operation (26.7%), a greater percent than general ATAAD postoperative mortality (18%) (19), suggested higher TSH may increase postoperative mortality. Cox regression analyses revealed that high-TSH SCH had a strong association with both 30-day mortality (HR 6.3, 95%CI 1.8-22.4, P=0.004) and postoperative mortality (HR 3.2, 95%CI 1.4-7.5, P=0.007). After adjusting for age, sex, and BMI, hypertension, ejection fraction, diabetes and history of PCI, the SCH with TSH ≥10 mU/L was still significantly associated with 30-day mortality (HR 6.2, 95%CI 1.7-22.0, P=0.005) and postoperative mortality (HR 3.4, 95%CI 1.4-8.0, P=0.005).

The mechanism of the relationship of preoperative SCH and ATAAD surgical mortality is unclear, but TSH itself has been suggested to stimulate the proliferation of vascular smooth muscle cells, leading to endothelial cell damage and inflammation and causing atherosclerosis (18). Marfella et al. found that atherosclerotic plaques in patients with SCH showed increased numbers of inflammatory cells and molecules, endothelial dysfunction markers, and decreased collagen content, which represent declining plaque instability (20). In addition, the elevated level of TSH and autoimmune reactivity may all contribute to reducing nitrogen monoxide, leading to the impairment of smooth muscle relaxation (21). Some evidence suggested that TSH induced interleukin-6 and tumor necrosis factor-α secretion *in vitro*, and TSH can increase nitrogen monoxide metabolites *in vivo*, which mediate inflammation and oxidative stress (15). Hypothyroidism may affect myocardial structure and function by impairing cardiac contractility and diastolic function and slowing left ventricular relaxation rate, which could harm ventricular filling during physical activities (22). Thus, all these alterations in vascular function and myocardial function may make the postoperative situation more critical, increasing mortality of ATAAD patients.

In the subgroup analysis, the effect of TSH on mortality was more pronounced in the lower FT4 cohort. During the critical illness, the peripheral conversion rate of FT4 may alter in this excessive stress state, and FT4 concentration declines because of its shorter half-life in the circulation compared with FT3 (23). Nagy et al. reported that the occurrence of low FT4 might signify an even worse physiological and neuroendocrine status among patients, and low FT4 probably acts as a marker of a critical state in which the thyroid gland has lost its remaining precursors (24). Thus, elevated TSH, along with reduced FT4, may predict worse outcomes of ATAAD patients. Besides, the effect of TSH on 30-day mortality was more pronounced in the lower FT3 subgroup. Previous studies have shown that low FT3 level was reported to be associated with higher mortality, increased length of hospitalization and a higher rate of ICU admission, even though its pathophysiological role is not well understood (25). However, there was a significantly increased postoperative mortality risk of the high TSH group in the subgroup of high FT3, not in the subgroup of lower FT3. This may be due to the effect of low FT3 on mortality is the result of short-term stress, but in the long-term follow-up, various risk factors, such as stroke and malignant tumor, as well as insufficient sample size, may interfere the effect of FT3 on death.

The requirement for perioperative thyroid drug intervention in ATAAD patients with SCH remains undetermined. But a consensus statement of three U.S. societies recommended treatment for patients with TSH concentration greater than 10 mU/L (26). In addition, according to the American Thyroid Association and the American Association of Clinical Endocrinologists, patients with cardiovascular disorders might benefit from earlier therapy (27). Zhang et al. found that thyroid replacement therapy can prevent major adverse cardiovascular events after PCI in SCH patients (28). Although the mechanism has not been thoroughly studied, it might be the result of negative feedback regulation through the hypothalamic-pituitary-thyroid axis (29). Therefore, thyroxine supplementation for ATAAD patients who have high-TSH SCH is reasonable (27). Plasma TSH concentration may decrease by exogenous thyroid hormone by negative feedback regulation, reducing its harmful effects on the cardiovascular system and avoiding the occurrence of adverse events. Razvi et al. (30) suggested that treating SCH by T4 replacement reduced the risk of CVD events and mortality. Oral L-thyroxine is the first choice of treatment because of its efficacy and safety; the therapy target is maintenance of TSH concentration within the reference range (31). In summary, more studies are needed to determine the impact of thyroxine therapy on the outcomes of ATAAD patients who have high-TSH SCH.

There are several limitations in our study. First, the study is a single center, retrospective investigation, and the evidence level is lower than that in multi-center, prospective studies. Second, we enrolled a relatively small sample of patients, and we do not have enough patients with normal range TSH as normal controls, a situation which might not reflect the real thyroid function of all ATAAD patients. Third, in the multivariable Cox regression, we adjusted for only age, sex, BMI, hypertension, ejection fraction, diabetes and history of PCI; we did not include other possible influencing factors. Fourth, although it is not likely that ATAAD affected serum TSH concentrations, the exposure-mediator association was not clear, and we need to consider the possibility of reverse causation.

To our knowledge, our study is the only cohort study on the relationship between preoperative SCH and ATAAD prognosis, and the study will provide the basis for more clinical and mechanistic research. Thyroid hormone should be used as a routine test indicator before surgery to assess the prognosis of ATAAD patients, but whether thyroxine therapy is required still needs further study such as randomized controlled trials.

## Conclusion

Preoperative high-TSH subclinical hypothyroidism is an independent predictor of 30-day mortality and postoperative mortality in ATAAD patients who undergo aortic surgery.

## Data Availability Statement

The raw data supporting the conclusions of this article will be made available by the authors, without undue reservation.

## Ethics Statement

The studies involving human participants were reviewed and approved by the Ethics Committee of Beijing Anzhen Hospital (Institutional Review Board File 2014019). Written informed consent for participation was not required for this study in accordance with the national legislation and the institutional requirements.

## Author Contributions

H-YL, W-JJ, and H-JZ contributed to conception and design of the study. H-YL, W-JJ, and H-JZ contributed to administrative support. W-JJ provided the study materials and patients. S-PW and YX contributed to data collection and data analysis. S-PW and YX wrote the first draft of the manuscript. All authors contributed to manuscript revision, read, and approved the submitted version.

## Funding

This study was supported by the National Natural Science Foundation of China (NO. 82170487 and NO. 82070483), Scientific Research Common Program of Beijing Municipal Commission of Education (NO. KM202110025014), Beijing Hospitals Authority Clinical medicine Development of special funding support (NO.XMLX202107), Beijing Municipal Science & Technology Commission (Z211100002921010), the Foundation of Beijing Outstanding Young Talent Training Program (No. 2017000021469G254).

## Conflict of Interest

The authors declare that the research was conducted in the absence of any commercial or financial relationships that could be construed as a potential conflict of interest.

## Publisher’s Note

All claims expressed in this article are solely those of the authors and do not necessarily represent those of their affiliated organizations, or those of the publisher, the editors and the reviewers. Any product that may be evaluated in this article, or claim that may be made by its manufacturer, is not guaranteed or endorsed by the publisher.
